# Supplementary material for: A chromosome-level genome assembly of the South African indigenous, Kolbroek pig, Sus scrofa domesticus
Source: Sci Data. 2026 Mar 9;13:635. doi: 10.1038/s41597-026-07002-y (PMC13100151; doi:10.1038/s41597-026-07002-y)
Supplement: Supplementary file 1 — Supplimentary information [file 41597_2026_7002_MOESM1_ESM.docx]

**Supplementary information**

**Supplementary Table 1**: A summary of the assembly pipeline that was used, along with versions available on <https://galaxy.eu>. The table also indicates which VGP workflow and version where available, was used to track galaxy-associated information.

| **VGP1** | **v0.1.7** |  |  |
| --- | --- | --- | --- |
| step | Tool | version | Reference |
| **K-mer analysis** | meryl | 1.3+galaxy6 | ^1^ |
|  | genomescope | 2.0.1+galaxy0 | ^2^ |
| **VGP 4** | **V0.2.2** |  |  |
| step | Tool | version | Reference |
| **Contig assembly** | cutadapt | 4.9+galaxy1 | ^15^ |
|  | multiqc | 1.11+galaxy1 | ^16^ |
|  | hifiasm | 0.19.9+galaxy0 | ^3^ |
|  | gfastats | 1.3.6+galaxy0 | ^4^ |
|  | ggplot2_point | 3.4.0+galaxy1 | ^5^ |
|  | busco | 5.5.0+galaxy0 | ^6^ |
|  | merqury | 1.3+galaxy4 | ^13^ |
| **VGP6** | **v0.4** |  |  |
| step | Tool | version | Reference |
| **Purge Duplicates** | minimap2 | 2.28+galaxy0 |  |
|  | purge_dups | 1.2.6+galaxy0 | <https://github.com/dfguan/purge_dups> |
|  | busco | 5.5.0+galaxy0 |  |
|  | gfastats | 1.3.6+galaxy0 |  |
|  | merqury | 1.3+galaxy4 |  |
|  | ggplot2_point | 3.4.0+galaxy1 |  |
| **VGP8** | **v0.2.8** |  |  |
| **step** | **Tool** | **version** | Reference |
| **Scaffold assembly** | gfastats | 1.3.6+galaxy0 |  |
|  | bwa_mem2 | 2.2.1+galaxy1 | ^7^ |
|  | bellerophon | 1.0+galaxy1 | ^8^ |
|  | pretext_map | 0.1.9+galaxy1 | <https://github.com/sanger-tol/PretextMap> |
|  | YaHS | 1.2a.2+galaxy2 | ^9^ |
|  | pretext_snapshot | 0.0.3+galaxy2 | <https://github.com/sanger-tol/PretextSnapshot> |
|  | busco | 5.5.0+galaxy0 |  |
|  | datamash | 1.8+galaxy0 |  |
|  | bedtools_bamtobed | 2.31.1+galaxy0 | ^24^ |
|  | ggplot2_point | 3.4.0+galaxy1 |  |
|  |  |  |  |
|  |  |  |  |
|  |  |  |  |
| **VGP9** | **v0.3** |  |  |
| **step** | **Tool** | **version** |  |
| **Decontamination** | kraken2 | 2.1.3+galaxy1 | ^10^ |
|  | gfastats | 1.3.6+galaxy0 |  |
| **step** | **Tool** | **version** |  |
|  |  |  |  |
| Colinearity | D-genies* |  | ^17^ |
| Summary | Blobtools | 4.0.7+galaxy2 | ^18^ |
| Summary | BlobToolKit | 4.0.7+galaxy2 |  |
| Manual Curation | PretextView* | 0.04 | <https://github.com/sanger-tol/PretextView> |
|  | YaHS | 1.2a.2+galaxy2 |  |
| Annotation | RepeatMasker | 4.1.5+galaxy0 |  |
| Annotation | Repeat Modeler | 2.0.5+galaxy0 |  |
| Annotation | RepeatScout | v1.0.6 | ^11^ |
| Annotation | RECON | v1.5.0 | ^11^ |
| Annotation | TRF | v4.09 | ^12^ |
| Annotation | Tiberius* | v1.1.4 | ^13^ |
| Quality control | FastQC | 0.12.1 | ^11^ |
| Mitogenome assembly | MitoHiFi | 3 | ^14^ |

*not available on Galaxy at the time of analysis

| **A**  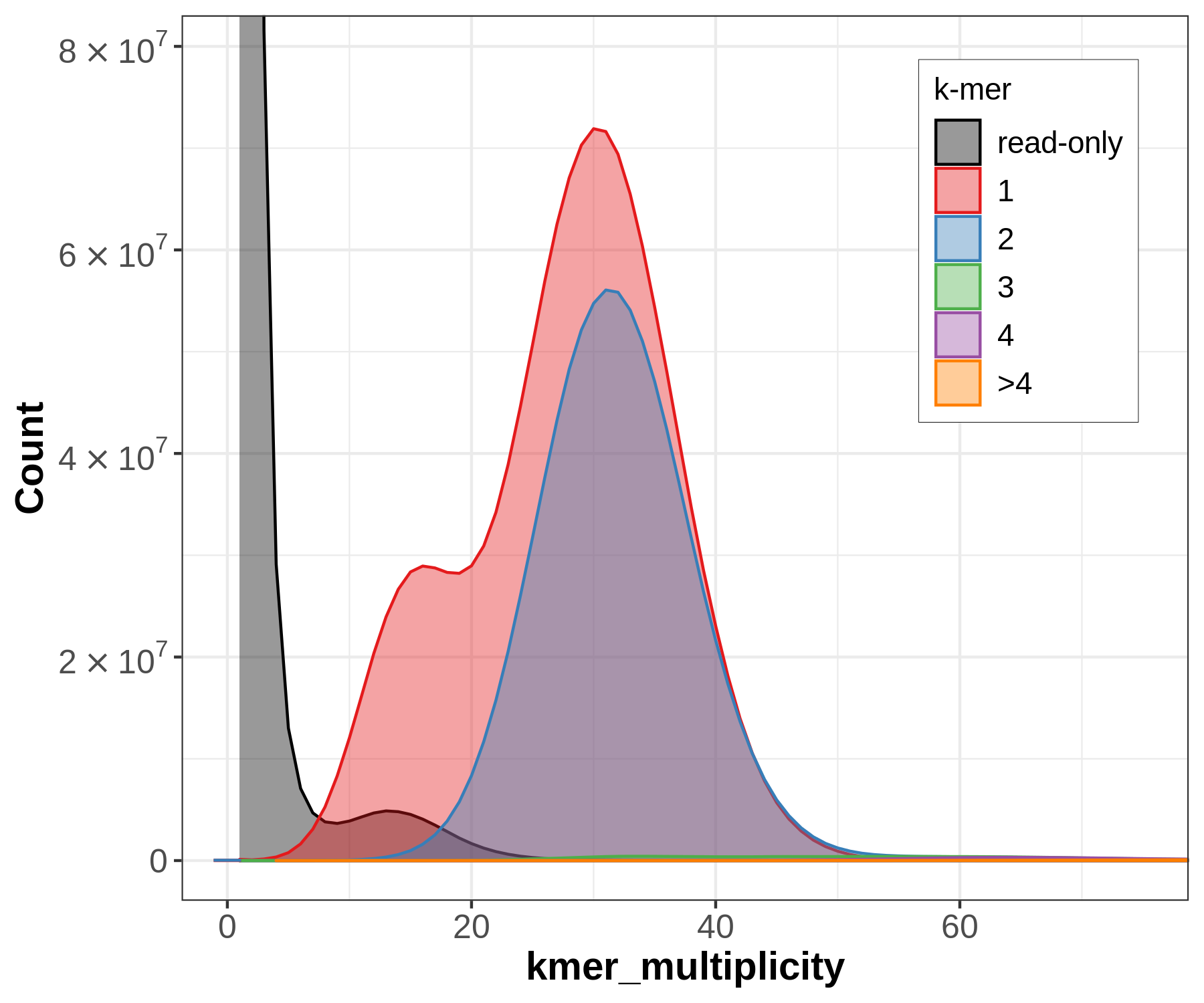 | **B**  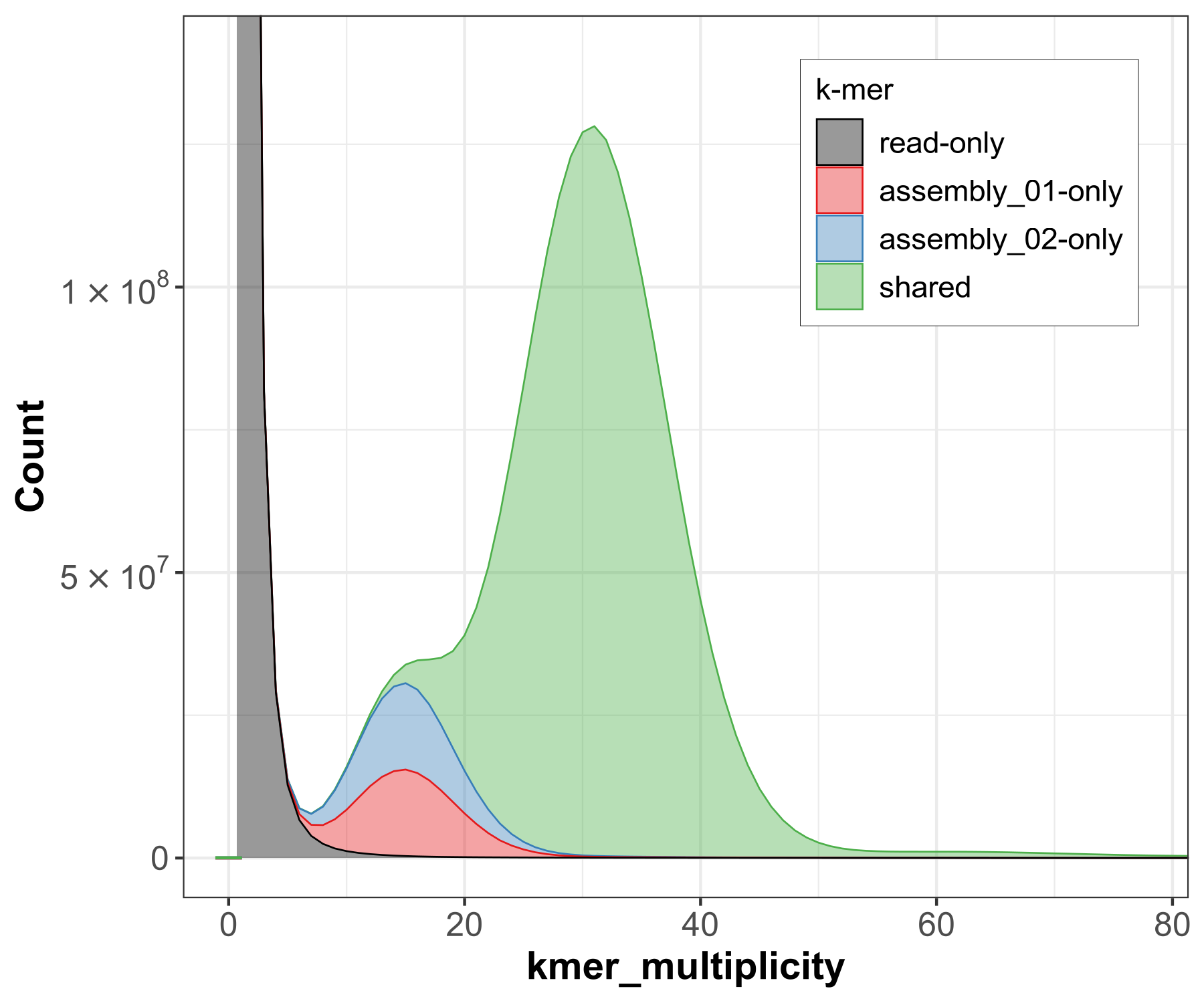 |
| --- | --- |

**Supplementary Figure 1**: A CN plot (A) that was generated for the hap 1 assembly showing the level of K-mer multiplicity for HiFi reads and an Assembly spectrum (ASM) plot (B) showing the K-mers identified, which are unique for each assembly at the contig level, as well as those that are shared.


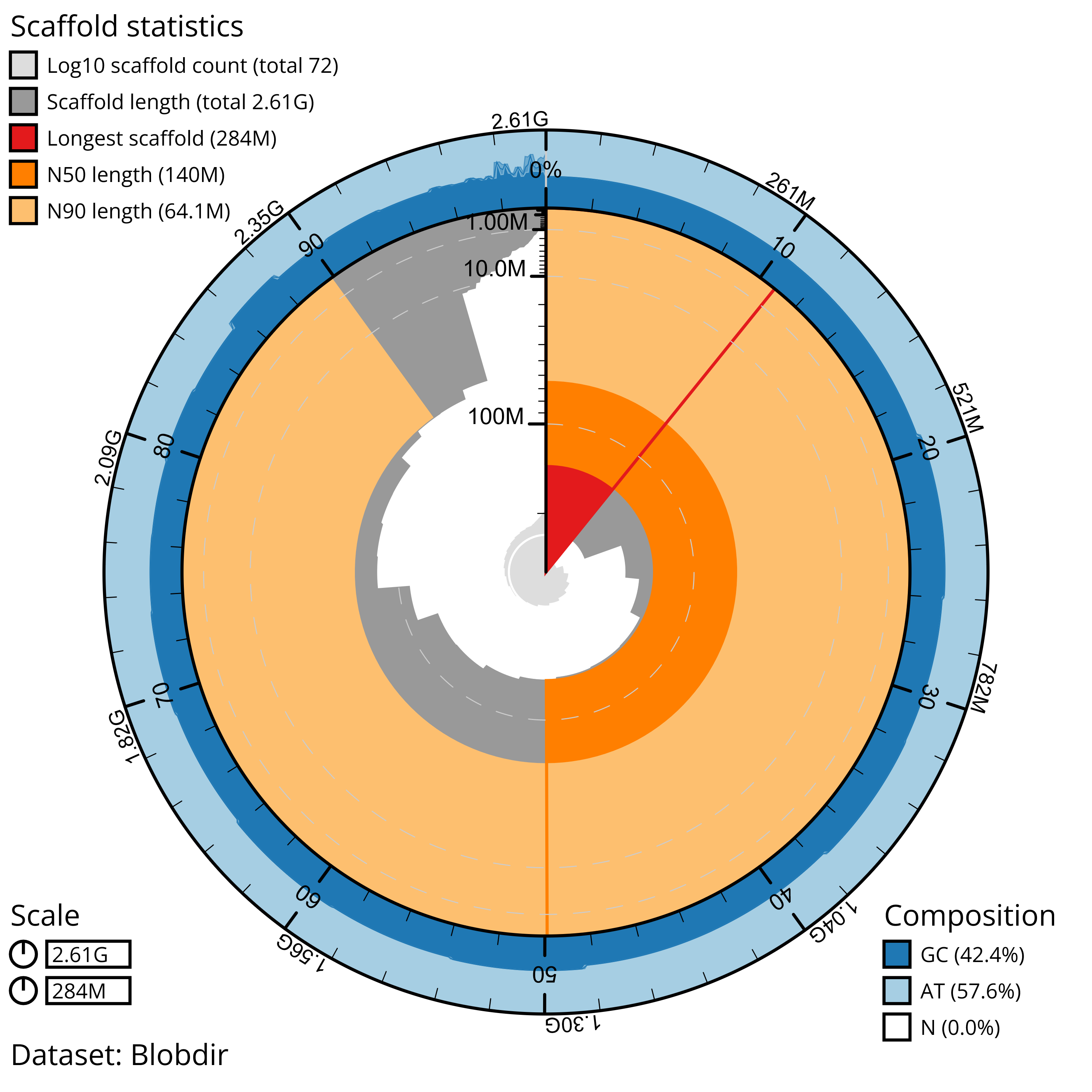


**Supplementary Figure 2**. A snail plot that graphically shows the quality of the primary genome assembly. The plot of the circumference of the circle represents the size (bp) of the genome, with the inner spiral describing the cumulative level of representation of each of the scaffolds. Dark red represents the first and longest scaffold, dark orange shows the scaffold N50 value, and light orange shows the included data for scaffold N90. The dark and light blue values show the percent GC and AT content respectively^15^.

| 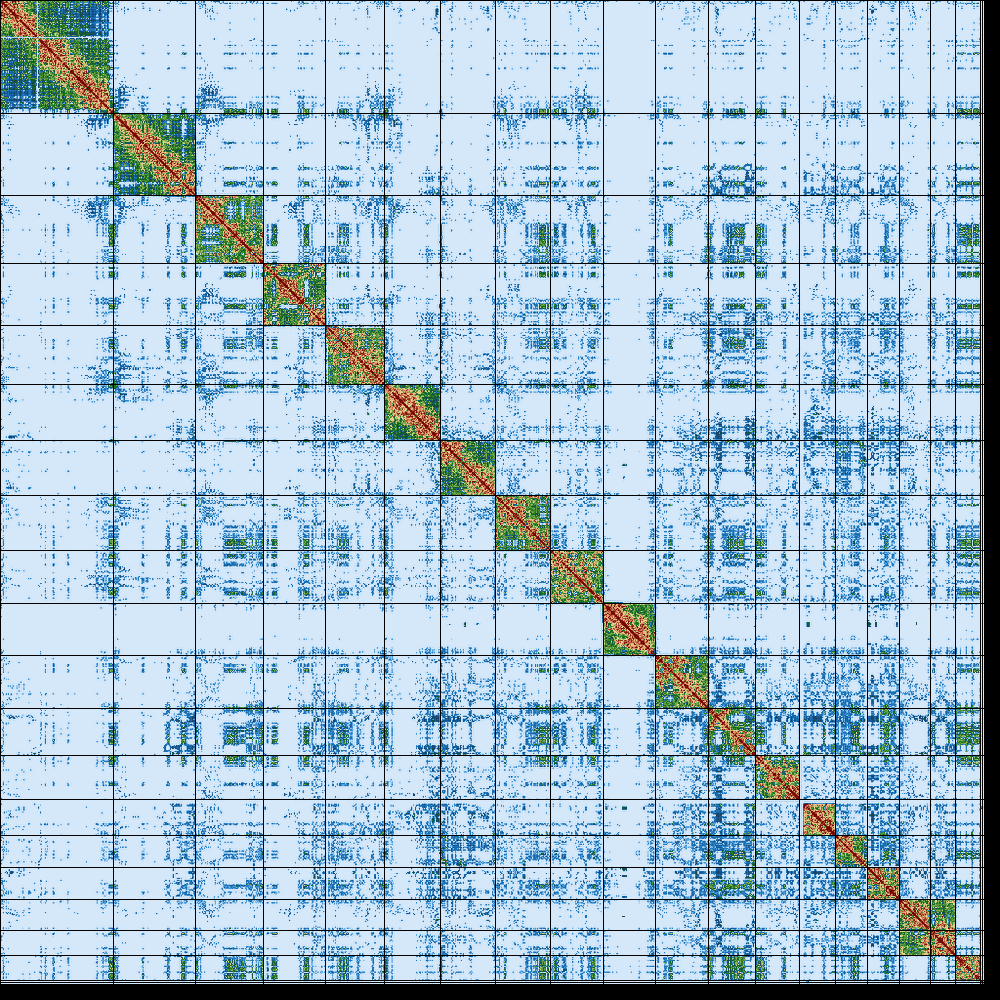 |
| --- |

**Supplementary Figure 3:** An Omni-C contact map generated against the Kolbroek primary assembly was generated, showing the chromosome-level scaffolds. The deeper intensity colour from light blue to dark red indicates the strength of the interactions between Omni-C reads and the assembly sequences. The scaffolds were ordered by size.

**Supplementary Table 2**: The results from the telomere analysis using the seqtk_telo application. The table displays the start and end regions, telomere lengths, as well as the average length for those that are identified. The bold Scaffold names indicate those that aligned to the reference during the Collinearity plot.

| **Chromosome** | **Start** | **End** | **Telomere length** |
| --- | --- | --- | --- |

| **scaffold_01** | 0 | 10249 | 10249 |
| --- | --- | --- | --- |
| **scaffold_01** | 284068673 | 284075074 | 6401 |
| **scaffold_02** | 0 | 10432 | 10432 |
| **scaffold_02** | 222666662 | 222672346 | 5684 |
| **scaffold_05** | 0 | 3774 | 3774 |
| **scaffold_06** | 142879150 | 142883766 | 4616 |
| **scaffold_07** | 0 | 4990 | 4990 |
| **scaffold_07** | 142073233 | 142083538 | 10305 |
| **scaffold_08** | 139841255 | 139850298 | 9043 |
| **scaffold_09** | 0 | 3970 | 3970 |
| **scaffold_09** | 136850103 | 136854792 | 4689 |
| **scaffold_11** | 0 | 12397 | 12397 |
| **scaffold_12** | 0 | 3090 | 3090 |
| **scaffold_14** | 80007280 | 80009781 | 2501 |
| **scaffold_17** | 0 | 803 | 803 |
| **scaffold_17** | 68683873 | 68687960 | 4087 |
| **scaffold_19** | 0 | 1561 | 1561 |
| **scaffold_20** | 0 | 6744 | 6744 |
| scaffold_23 | 0 | 5250 | 5250 |
| scaffold_27 | 6018641 | 6020598 | 1957 |
| scaffold_33 | 0 | 2545 | 2545 |
| scaffold_35 | 0 | 4536 | 4536 |
| scaffold_72 | 18730 | 28028 | 9298 |
| Mean length |  |  | 5605.30 |

References

1. Rhie, A., Walenz, B. P., Koren, S. & Phillippy, A. M. Merqury: reference-free quality, completeness, and phasing assessment for genome assemblies. *Genome Biology* **21**, 1–27 (2020).

2. Ranallo-Benavidez, T. R., Jaron, K. S. & Schatz, M. C. GenomeScope 2.0 and Smudgeplot for reference-free profiling of polyploid genomes. *Nat Commun* **11**, 1432–1432 (2020).

3. Haoyu Cheng, Gregory T. Concepcion, Xiaowen Feng, Haowen Zhang & Heng Li. Haplotype-resolved de novo assembly using phased assembly graphs with hifiasm. *Nature Methods* **18**, 170–175 (2021).

4. Formenti, G. *et al*. Gfastats: conversion, evaluation and manipulation of genome sequences using assembly graphs. *Bioinformatics* **38**, 4214–4216 (2022).

5. Wickham, H. in *ggplot2 : Elegant Graphics for Data Analysis* (Springer New York, New York, NY, 2009).

6. Mosè Manni, Matthew R Berkeley, Mathieu Seppey, Felipe A Simão & Evgeny M Zdobnov. BUSCO Update: Novel and Streamlined Workflows along with Broader and Deeper Phylogenetic Coverage for Scoring of Eukaryotic, Prokaryotic, and Viral Genomes. *Molecular Biology and Evolution* **38**, 4647–4654 (2021).

7. Vasimuddin, M., Misra, S., Li, H. & Aluru, S. *Efficient Architecture-Aware Acceleration of BWA-MEM for Multicore Systems*, IEEE, May 2019).

8. Huber, T., Faulkner, G. & Hugenholtz, P. Bellerophon: a program to detect chimeric sequences in multiple sequence alignments. *Bioinformatics* **20**, 2317–2319 (2004).

9. Zhou, C., McCarthy, S. A. & Durbin, R. YaHS: yet another Hi-C scaffolding tool. *Bioinformatics* **39** (2023).

10. Derrick E. Wood, Jennifer Lu & Ben Langmead. Improved metagenomic analysis with Kraken 2. *Genome Biology* **20**, 1 (2019).

11. Bao, Z. & Eddy, S. R. Automated De Novo Identification of Repeat Sequence Families in Sequenced Genomes. *Genome Research* **12**, 1269–1276 (2002).

12. G. Benson. Tandem repeats finder: a program to analyze DNA sequences. *Nucleic Acids Research* **27**, 573–580 (1999).

13. Gabriel, L., Becker, F., Hoff, K. J. & Stanke, M. Tiberius: end-to-end deep learning with an HMM for gene prediction. *Bioinformatics (Oxford, England)* **40** (2024).

14. Uliano-Silva, M. *et al*. MitoHiFi: a python pipeline for mitochondrial genome assembly from PacBio high fidelity reads. *BMC bioinformatics* **24**, 288–13 (2023).

15. Challis, R., Richards, E., Rajan, J., Cochrane, G. & Blaxter, M. BlobToolKit – Interactive Quality Assessment of Genome Assemblies. *G3 : genes - genomes - genetics* **10**, 1361–1374 (2020).
